# Subtle structural abnormalities in genotype positive phenotype ‘negative’ patients with pre-clinical hypertrophic cardiomyopathy (HCM): a blinded, controlled cardiovascular magnetic resonance (CMR) study

**DOI:** 10.1186/1532-429X-14-S1-O96

**Published:** 2012-02-01

**Authors:** Djeven Deva, Christiane Gruner, Melanie Care, Bernd J Wintersperger, Harry Rakowski, Andrew M Crean

**Affiliations:** 1 Department of Medical Imaging, University Health Network, Toronto, ON, Canada; 2 Department of Cardiology, University Health Network, Toronto, ON, Canada

## Summary

To evaluate the utility of cardiovascular magnetic resonance (CMR) in detection of individuals with pathogenic/ presumed pathogenic (PA/ PP) sarcomeric mutations and gene negative patients with high clinical probability of HCM but with a negative phenotype by standard diagnostic criteria.

## Background

Mutation carriers without hypertrophy - gene positive, phenotype negative (G+P-) - are not uncommon in clinical practice. There is debate about the presence of subtle abnormalities or variants of the left ventricle in G+P- individuals. Previous research into this area only evaluated limited mutations in the myosin binding protein C and α-tropomyosin genes. We hypothesized: 1) that G+P- patients with pathogenic or presumed pathogenic (PA/PP mutations) have subtle abnormalities of LV morphology recognisable by CMR in pre-clinical HCM but absent in a control population; and 2) that these signs are also present in gene negative patients with a normal wall thickness (G-P-) but high clinical probability of HCM (based on assessment of clinical or family history, electrocardiographic or echocardiographic criteria).

## Methods

The study population had 3 groups - Group 1 (G+P-, tested positive for PA/ PP mutations, n=18); Group 2 (G-P-, Under evaluation for HCM, tested negative for [PA/ PP] mutations, n=20); and Group 3 (controls, n=36). Two experienced CMR readers (blinded to results of genetic testing and clinical context) scored each case for probability of the case representing HCM based on pre-defined signs at CMR including the presence/absence of: a) deep basal inferoseptal crypts (DBISC); b) sudden wall thickness change by >50% in adjacent segments (SWTC); c) inappropriate wall thinning (IWT); d) apical-basal false tendon (ABFT); e) lack of apical tapering (LAT); f) snub nose apical contour (SNAC) . Cases were reviewed individually and in consensus. An experienced HCM cardiologist (blinded to CMR findings) reviewed clinical data, electrocardiograms and echocardiograms and scored each case for likelihood of HCM.

## Results

Of the 18 G+P- patients, 78% had DBISC, 67% had an ABFT, 50% had IWT and 39% had SWTC. DBISC were seen in cases with PA/PP MYBPC3, TPM1, TNNT2 and MYH7 mutations. All G+P- cases had at least one subtle sign. All G-P- cases classified clinically as high probability of HCM, were classified as high probability of HCM on imaging grounds and had 2 or more subtle signs of HCM: DBISC, SWTC, IWT, ABFT, SNAC and LAT. Controls had no DBISC, SWTC, IWT or LAT.

## Conclusions

Subtle structural abnormalities visible on CMR help identify PA/PP sarcomeric mutation carriers, as well as G- P- with high clinical probability of HCM, in a genetically heterogenous population.

## Funding

None.

**Table 1:** Incidence of Subtle Signs in G+P- Group and Controls

Sign	G+P- Group (n=18)	Controls (n=36)
DBISC	14 (77.8%)	0 (0%)
SWTC	7 (38.9%)	0 (0%)
IWT	9 (50%)	0 (0%)
ABFT	12 (66.7%)	2 (5.6%)
LAT	1 (5.6%)	0 (0%)
SNAC	1 (5.6%)	1 (2.8%)

(DBISC - Deep Basal Inferoseptal Crypts, SWTC - Sudden Wall Thickness Change, IWT - Inappropriate Wall Thinning, ABFT - Apical to Basal False Tendon, LAT - Lack of Apical Tapering, SNAC - Snub Nose Apical Contour). DBISC are defined as focal defects in the basal inferoseptum with a depth of greater than 50% relative to immediate surrounding myocardium. SWTC is defined as sudden transition in wall thickness between adjacent myocardial segments with a ratio of thicker to thinner myocardium of 2:1 or greater (excluding the thin myocardium of the true apex) IWT is defined as wall thinning out of keeping with the thickness of adjacent myocardial segments or segments within the ring of myocardium that segment is located in. ABFT is defined a false tendon extending from the basal ventricle to apical compacted myocardium. LAT is defined as apical myocardial tapering not conforming to normality (typically the myocardium tapers smoothly towards the apex becoming progressively thinner till it reaches the thin myocardium of the tip of the true apex. SNAC is defined as a flattened or blunted epicardial contour in systole akin to the shape of the tip of a ballet dancer’s shoe.

**Figure 1:**
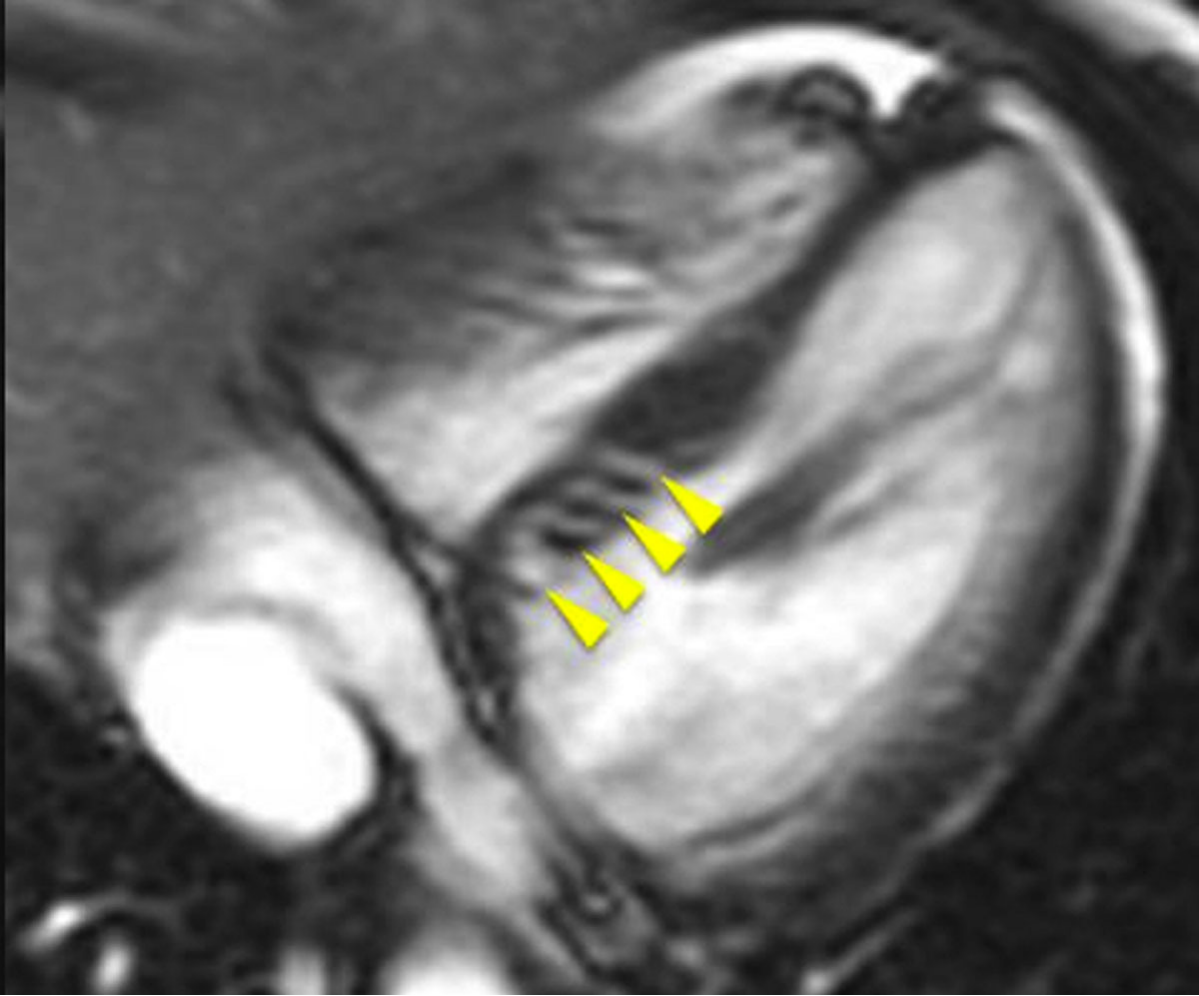
Basal 4 chamber orientation image from an SSFP cine stack demonstrating multiple deep basal inferoseptal crypts (DBISC) in a 20 year old female with a pathogenic p.Glu163del TNNT2 mutation.

